# Does the registry speak your language? A case study of the Global Angelman Syndrome Registry

**DOI:** 10.1186/s13023-023-02904-1

**Published:** 2023-10-19

**Authors:** Megan Tones, Nikolajs Zeps, Yvette Wyborn, Adam Smith, Roberto A. Barrero, Helen Heussler, Meagan Cross, James McGree, Matthew Bellgard

**Affiliations:** 1 Office of eResearch, Queensland University of Technology, Brisbane, QLD 4000 Australia; 2 Centre for Clinical Trials in Rare Neurodevelopmental Disorders, Child Development Program, Children’s Health Queensland, Child Health Research Centre University of Queensland, Brisbane, QLD 4101 Australia; 3 Foundation for Angelman Syndrome Therapeutics Australia, Salisbury, QLD 4107 Australia; 4 School of Mathematical Sciences, Queensland University of Technology, Brisbane, Australia; 5 University of East London, London, UK

**Keywords:** Disease Registry, Patient reported outcomes, Language translation, Equity diversity and inclusion

## Abstract

Global disease registries are critical to capturing common patient related information on rare illnesses, allowing patients and their families to provide information about their condition in a safe, accessible, and engaging manner that enables researchers to undertake critical research aimed at improving outcomes. Typically, English is the default language of choice for these global digital health platforms. Unfortunately, language barriers can significantly inhibit participation from non-English speaking participants. In addition, there is potential for compromises in data quality and completeness. In contrast, multinational commercial entities provide access to their websites in the local language of the country they are operating in, and often provide multiple options reflecting ethnic diversity. This paper presents a case study of how the Global Angelman Syndrome Registry (GASR) has used a novel approach to enable multiple language translations for its website. Using a “semi-automated language translation” approach, the GASR, which was originally launched in English in September 2016, is now available in several other languages. In 2020, the GASR adopted a novel approach using crowd-sourcing and machine translation tools leading to the availability of the GASR in Spanish, Traditional Chinese, Italian, and Hindi. As a result, enrolments increased by 124% percent for Spain, 67% percent for Latin America, 46% percent for Asia, 24% for Italy, and 43% for India. We describe our approach here, which we believe presents an opportunity for cost-effective and timely translations responsive to changes to the registry and helps build and maintain engagement with global disease communities.

## Background

Angelman syndrome (AS) is a severe neurodevelopmental disorder caused by dysfunction of the maternally inherited UBE3A gene. It is estimated that 500,000 people live with AS worldwide [1]. Global rare disease registries are a valuable tool for enhancing therapeutics in rare diseases, enabling participant recruitment and capture and monitoring of patient reported outcomes, amongst other uses. Despite the need to be Global, there is a lack of diversity in terms of language available on global registry websites. This is, unfortunately, common in medicine and science where the fact that the common scientific language is English has spilled over into an apparent insistence that participants in research from non-English speaking countries must be done in English. In contrast, no multinational commercial entity would survive if it took this approach and as a result, they are available in myriad local languages. For Example, the website for the global movement Rare Disease Day is available in 103 languages besides English [2], while the European Commission websites strive to be available in all 24 recognised European languages [3]. Registry development guidelines including the fourth edition of the guide *Registries for Evaluating Patient Outcomes* released by the Agency for Healthcare Research and Quality [4] and Rare Diseases Registry Program [5], stress the importance of careful translation of multinational registries. Other international registries such as the Hyperinsulinism Global Registry or Global Prader Willi Registry are intending to incorporate multiple languages [6, 7]. Another strategy is to establish a federation of linked registries for a rare disease to achieve global coverage of patients [8]. Towards this end, the Global Angelman Syndrome Registry is open to data linkages with other data sets including Natural History Studies and registries such as the Angelman Syndrome Online Registry [9]. Barriers to making services multilingual include lack of access to translators, and the need for technical expertise or an understanding of the topic of the registry. Tools such as google translate have demonstrated that technology can be used to assist with this, although native speaker input is still required to ensure accuracy and readability of translations.

Findings from a review of articles on methodological approaches to the cross-cultural adaptation of surveys and tools indicated that translators should be fluent in the source and target languages, understand both cultures, and knowledgeable about the content of the instrument being adapted [10]. Addressing each of these requirements may be challenging, as professional translators may not be subject matter experts and will lack specialised content knowledge. Involving more than one translator in the process may be beneficial to offer a mix of perspectives with respect to language fluency, cultural understanding, and content knowledge. However, this may prove difficult due to challenges around document sharing version control, and managing division of workload inhibiting translator interactions. Additionally, reconciliation and review of translations by an expert panel, and cognitive interviews or pilot testing with focus groups should be undertaken to determine the face and content validity of translated instruments [10]. There is limited evidence the value of back translations [10].

### Use of technology to facilitate translations

*Machine translation* (MT) involves using software tools to translate text or speech from the source language to the target language [11]. The process is automated and may involve different approaches including rules created by linguists and computer scientists, examples from a database of source and target language sentences, and statistical modelling of the probability that a target sentence is the correct translation of a source sentence [12].

*Translation memories* (TM) are a related technology which involves storing previously completed human translations, including the source text and translated text, in a database and matching segments of text, such as a sentence, from the TM database with new source text to create translations [13]. Matches may be exact, or identical including formatting; full, with differences such as numbers or dates; or fuzzy, which is similar but requires editing [13].

*Crowdsourcing* refers to an organisation (such as a research institution or not for profit) outsourcing a task previously undertaken internally to an external community to complete a task or solve a problem for mutual benefit [14]. In research, crowdsourcing has been used for a range of tasks including identification and classification, transcription or translation, and data collection and analysis [15]. Organisations including Cochrane and Technology, Entertainment and Design (TED) talks involve volunteer translators to translate resources in recognition of the fact that most people globally do not speak English as a first language [16].

Rare disease registries may present a unique opportunity for crowdsourcing translations, as rare disease communities often drive the development of registries and have strong involvement in registry governance and ownership. While crowdsourcing may seem advantageous in this context, projects must be managed effectively to prevent negative outcomes such as translator or researcher burnout or malicious translations. Blohm et al. [17] reviewed the management and governance of a variety of crowdsourcing projects and determined a four-step process for running a crowdsourcing project: (1) Define Goal and System Type; (2) Start Small and Experiment; (3) Build up Scalable Structures and (4) Adapt and Monitor Governance (p. 143).

### Current study

The Global Angelman Syndrome Registry was launched in English in September 2016 [18]. The registry was sponsored by the Foundation for Angelman Syndrome Therapeutics (FAST) Australia. The aims of the registry include:

1. Facilitate participant recruitment for clinical trials;
2. Collect the natural history of a large cohort of individuals with AS;
3. Identify demographic, phenotypic and genotypic variation in clinical features and outcomes; and
4. Aid in service provision planning for individuals with AS and their families.

The registry was originally deployed using the Rare Disease Registry Framework (RDRF) [18–33], an internet-based modular registry framework, developed by the Centre for Comparative Genomics at Murdoch University.

Since its launch, AS organisations have expressed an interest in translation into multiple languages. Initially, the translation process was to include three steps: (1) Forward translation; (2) Back translation; and (3) Pilot testing. The forward translation process was completed for Italian, Spanish, French and Hebrew, and partially completed for Portuguese and Chinese.

In 2020, the registry was moved to the Trial Ready Registry Framework (TRRF) [30, 32, 34–39]. The new platform incorporated significant revisions based on feedback from families, clinicians and researchers. Changes included revisions to both content and functionality to simplify the user experience in completing forms, enable longitudinal data collection and user managed linkages with clinicians and researchers, and integrate translations and analytics.

Due to changes to the registry content and function, and the likelihood of the requirement for updates for existing translations and the addition of more languages, alternative methodologies for translations were explored that were less burdensome on the community and research team. The current study reports on the establishment of the GASR translation project.

## Method

To facilitate timely translation of the GASR, *Crowdin* [40] was selected as a tool to integrate existing translations (converted to TM) and MT provided by *Crowdin* software with crowdsourcing translations from the Angelman community and manage the translation process.

### Registry description

The GASR features a series of forms, or modules that collect information on an individual with AS’s condition. The forms cover demographic, clinical, behavioural and developmental information. The content of the GASR modules and other patient facing information including the registration form, standard emails, and website messages constituted the information to be translated. There were approximately 13 000 words on the GASR across the 20 sections to be translated.

Prior to the availability of the translations, 1625 families have joined the registry, 1614 of whom had provided geographic data. As shown in Table 1, most families were from English speaking countries.

**Table 1:** Country/region of residence, families in the Angelman Registry Prior to Translation Availability

Country	N	%
United States	712	44.3
Australia	166	10.3
Canada	95	5.9
United Kingdom	89	5.5
Brazil	65	4.0
Germany	42	2.6
Poland	42	2.6
Italy	41	2.5
Spain	37	2.3
Chile	29	1.8
Western Asia	23	1.4
Colombia	22	1.4
Greece	21	1.3
India	21	1.3
France	18	1.1
New Zealand and the Pacific	16	1.0
Russia	16	1.0
Argentina	14	0.9
Latin America and the Carribbean	14	0.9
Northern Europe	13	0.8
Western Europe	13	0.8
Netherlands	12	0.7
Southern Europe	11	0.7
Eastern Europe	11	0.7
Eastern Asia	10	0.6
Ireland	10	0.6
Mexico	9	0.6
Northern Africa	8	0.5
Hungary	6	0.4
Portugal	6	0.4
South Africa	6	0.4
South-eastern Asia	5	0.3
Southern Asia	5	0.3
Total	1608	100

### Governance and management of crowdsourcing

A *Crowdin Enterprises* project, hereafter referred to as *Crowdin*, (https://eresearchqut.crowdin.com/) was established to manage the project. *Crowdin* allows for different levels of access to ensure that participants only have access to tasks to which they are assigned by the project administrator. As the registry was pre-translated, the volunteers were assigned one of two roles: community proofreader or final proofreader. Community proofreaders review and modify existing machine translations, while final proofreaders are trusted professionals from the AS community who review and correct community proofread translations.

Proofreaders access the registry content via a testing site located at https://trrf.qa.angelmanregistry.info/. The website enables users to view the translations in the context of the website and access the *Crowdin* editor tool for each string. The editor tool shows the source text and several translations obtained from translation memory files or machine translations. The proofreader can revise, add or approve translations, and leave comments for other project team members.

Community proofreaders participated in a small group training session with one of the authors (M.T.), who explained the workflow of the project and how to use the *Crowdin* tool. For the purposes of scalability, one language was selected for pilot testing and refining the translation process. To date, training sessions have been held with community proofreaders for the Italian, Spanish, Chinese, Portuguese and French languages. Hindi was subsequently translated by an external company capable of interfacing with *Crowdin.*

The author running the training sessions checked in with a nominated translator from each group weekly to receive updates about progress and obtain feedback.

### Research ethics

The registry team submitted ethics amendments to incorporate translations into the protocol. Approval was granted for the translation of registry materials using the methods described above from the Mater Health Services Human Research Ethics Committee (HREC/13MHS/76/ Project 20,865).

## Results

**Fig. 1:**
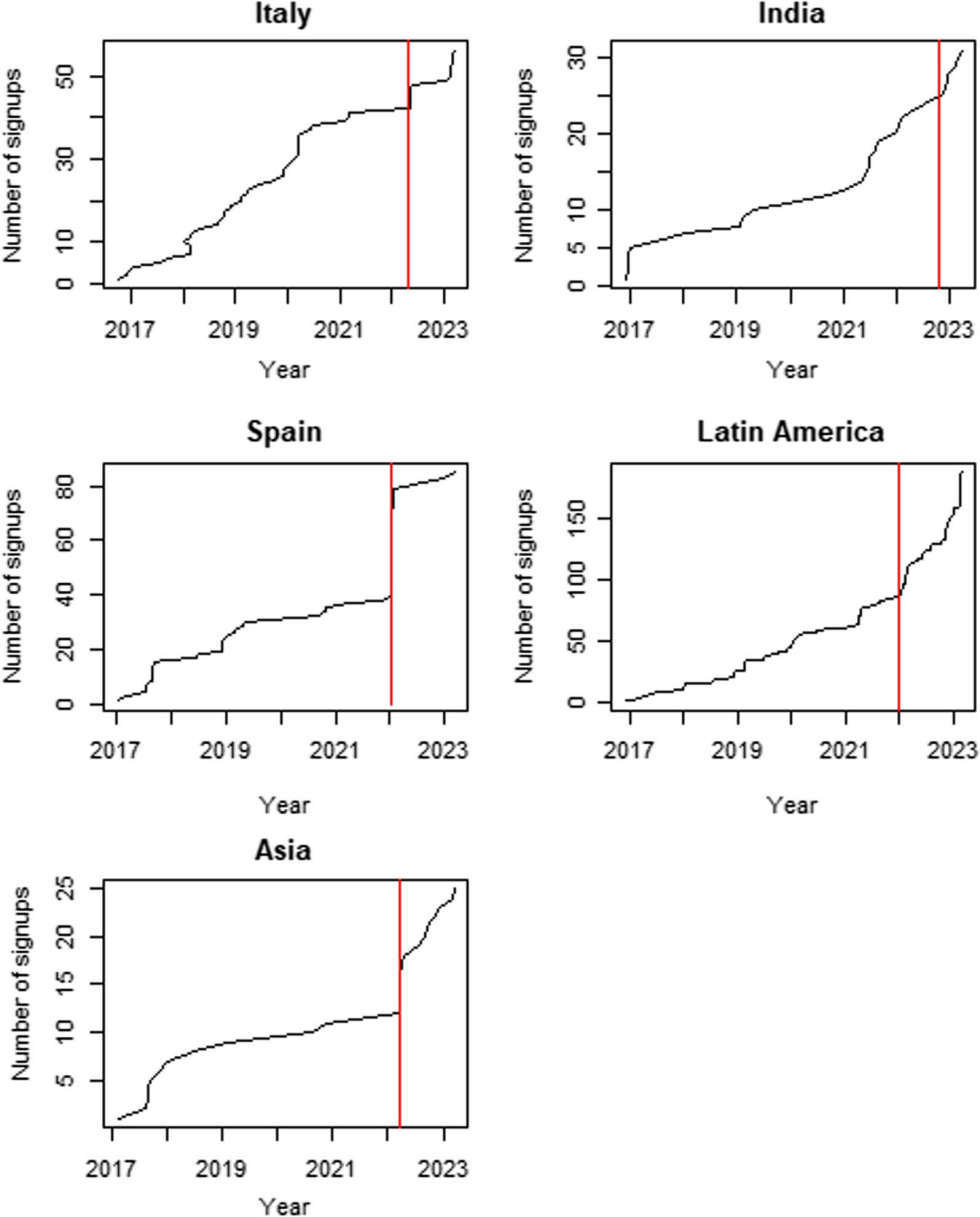
Growth in registry participation pre and post translations

The Spanish, Traditional Chinese, Italian and Hindi versions of the registry were launched in 2022 on the 6th January, 22nd March, 27th of April and 13th October respectively. Growth in registry enrolments post translations for each country or region where the language is spoken are shown in Fig. 1, along with current totals.

### Observations and feedback from the translation process

*The Crowdin tool was user friendly.* Proofreaders utilised a mix of the *Crowdin* editor tool and in-context editing tool. The editor tool displayed the source text and available translations side by side, enabling users to correct existing translations or add new translations. The *Crowdin* editor tool was the primary method used for proofreading, as all strings were displayed in the interfacing, ensuring comprehensive proofreading. The in-context editing tool was implemented on a test website which replicated the GASR site, which was valuable to view how the translations would appear to users in the context of the registry. A list of examples of source text, machine translations, and community and professional proofreading corrections is shown in Table 2.

**Table 2:** Example of source text with machine translations, and community and professional proofreading corrections

Source text	Machine translations	Community translations	Professional translations
*Italian*			
On a typical day, how many hours do they spend using the device for AAC purposes?	In una giornata tipica, quante ore trascorrono a utilizzare il dispositivo per scopi AAC?	In una giornata tipica, quante ore spendono usando il dispositivo per scopi CAA?	In una giornata tipica, per quante ore il bambino/adulto utilizza il dispositivo per la CAA?
Gross Motor Function—please describe your child/adult's ability to do the following:	Funzione motoria lorda: descrivi la capacità di tuo figlio/adulto di fare quanto segue:	Funzione grosso motoria—descrivere la capacità del paziente di fare quanto segue:	Abilità grosso motorie—descrivere la capacità del paziente di fare quanto segue:
Please report on your child/ adult's current seizure status	Si prega di riferire sullo stato attuale delle crisi epilettiche di suo figlio/adulto	Si prega di segnalare lo stato attuale del quadro epilettico del paziente	Si prega di segnalare lo stato attuale del quadro epilettico del paziente
Please tell us what medications/interventions your child/ adult is currently taking	Dicci quali farmaci/interventi sta attualmente assumendo tuo figlio/adulto	Per favore, indica quali farmaci o quali terapie sono attualmente somministrati al paziente	Per favore, indica quali farmaci o quali trattamenti sono attualmente somministrati al paziente
*Spanish*			
Adaptive Behaviour: Dressing—please describe your child/adult's ability to do the following:	Comportamiento adaptativo: cómo vestirse: describa la capacidad de su hijo/adulto para hacer lo siguiente:	Comportamiento Adaptativo: Vestirse- por favor describe la capacidad de su niño/adulto para hacer lo siguiente:	Comportamiento Adaptativo: Vestirse- por favor describa la capacidad de su niño/adulto para hacer lo siguiente:
For what function of communication does your child/ adult use AAC with you?	¿Para qué función de comunicación usa su hijo/adulto el AAC con usted?	¿Para qué función de comunicación su hijo/adulto utiliza CAA con usted?	¿Para qué función de comunicación su niño/adulto utiliza CAA con usted?
Individual with Angelman Syndrome's Results	Resultados de una persona con síndrome de Angelman	Resultados del Individuo con Síndrome de Angelman	Individuo con los resultados del Síndrome de Angelman
What was your child/ adult's age when medication/ intervention was started?	¿Cuál era la edad de su hijo o adulto cuando comenzó la medicación o la intervención?	¿Cuál era la edad de su niño/adulto cuando se inició la medicamento/intervención?	¿Cuál era la edad de su niño/adulto cuando se inició la medicación /intervención?
*Traditional Chinese*			
Would you describe your child/ adult’s gait as typical when compared to typically developing age matched peers?	與通常發展中的年齡匹配的同齡人相比, 您會將您的孩子/成人的步態描述為典型嗎?	與一般發展年齡相符的同齡人相比, 您會將您的孩子/患者的步態形容為典型嗎?	與相同發展年齡的一般人相比, 您會形容您的孩子/患者步態正常嗎?
How is the constipation (or regular bowel function) managed?	如何管理便秘 (或正常排便功能) ?	便秘(或定期腸功能)是如何管理的?	便秘(或定期排便功能) 是如何管理的?
How would you describe your child/ adults’ muscle tone?	你會如何形容你的孩子/成人的肌肉張力?	你會如何形容你的孩子/患者的肌肉活動?	你會如何形容你的孩子/患者的肌肉張力?
Has your child/ adult had any other medical conditions that have not been covered?	您的孩子/成人有沒有受保的其他醫療狀況?	您的孩子/患者有否其他未包括的醫療狀況?	您的孩子/患者有否其他未包括的疾病?
*Hindi*			
Is your child/ adult currently participating in Augmentative and Alternative Communication (AAC) therapy?	क्या आपका बच्चा/वयस्क वर्तमान में ऑगमेंटेटिव एंड अल्टरनेटिव कम्युनिकेशन (AAC) थेरेपी में भाग ले रहा है?		क्या आपका बच्चा/वयस्क वर्तमान में ऑगमेंटेटिव एंड अल्टरनेटिव कम्युनिकेशन (AAC) थेरेपी में भाग ले रहा है?
Were there any reflux/gastro/oesophageal problems?	क्या कोई रिफ्लक्स/गैस्ट्रो/ओसोफेगल समस्याएं थीं?		क्या कोई रिफ्लक्स/गैस्ट्रो/ओसोफेगल समस्याएं थीं?
Please comment on any other details about their tonic, clonic, or tonic–clonic seizures (eg significant seizure events/ changes)	कृपया उनके टॉनिक, क्लोनिक, या टॉनिक-क्लोनिक दौरे (जैसे महत्वपूर्ण दौरे की घटनाएं/परिवर्तन) के बारे में किसी भी अन्य विवरण पर टिप्पणी करें		कृपया उनके टॉनिक, क्लोनिक, या टॉनिक-क्लोनिक दौरे (जैसे महत्वपूर्ण जब्ती घटनाओं/परिवर्तन) के बारे में किसी भी अन्य विवरण पर टिप्पणी करें
What type of seizure was it?	यह किस प्रकार का दौरा था?		यह किस प्रकार की दौरा था?

A professional medical translation company was hired to complete the Hindi translation, therefore, the community translation column is blank for this language

### A group of at least three proofreaders was advantageous

Larger groups reduced the workload for individual proofreaders and enabled more comprehensive review of translations prior to the final proofreading step. For instance, the Spanish proofreading group identified that the word for “boy” and “child” was the same and could thus lead to Spanish speaking families reading sections of the registry as “boy/ adult” rather than “child/ adult.” The author in communication with the translation team (MT) was able to put them in contact with another author (RB) whose first language was Spanish.

### Preliminary validation findings are encouraging

Although validation is a separate step beyond translation, the authors compared 107 English and 55 Spanish responses to the *Newborn and Infancy* module completed since the translations were implemented. This module was selected as being the first module users encounter in the registry, it had the highest completion rate, and responses were thought to be less impacted by the age and genotype of the person with Angelman syndrome. Responses to Likert scale items from the Newborn and Infancy module are shown in Table 3. A series of 25 Chi square tests were conducted, with a Bonferroni adjustment indicating an adjusted alpha level of *p* = 0.002. Out of the 25 questions, only two demonstrated significant differences between the English and Spanish samples, reflecting that:

- Spanish speaking parents perceived their infant with Angelman syndrome to be placid more frequently than English speaking parents.
- English speaking parents perceived their infant with Angelman syndrome to experience more frequent reflux/gastro/oesophageal problems than Spanish speaking parents.

**Table 3:** Comparison of English and Spanish language responses to Newborn and Infancy module items

Questions	Language	1—Yes all the time		2—Yes, most of the time		3—Yes, some of the time		4—Yes, rarely		5—No, never		Unknown		Total	Chi square tests
Newborn questions		*n*	%	*n*	%	*n*	%	*n*	%	*n*	%	*n*	%	*N*	
Were feeding difficulties experienced?	English	35	33	29	27	19	18	8	8	15	14	0	0	106	*Χ*^2^ (4, 159) = 8.347, *p* = .08
	Spanish	12	23	10	19	8	15	9	17	14	26	0	0	53	
Was assistance used at any time in their infancy (eg lactation support, syringes, spooning in pumped milk)?	English	18	19	21	22	18	19	9	10	28	30	0	0	94	*Χ*^2^ (5, 137) = 4.279, *p* = .510
	Spanish	7	16	7	16	6	14	5	12	17	40	1	2	43	
Was there refusal to nurse?	English	19	20	9	10	24	26	7	8	29	31	5	5	93	*Χ*^2^ (5, 133) = 0.622, *p* = .987
	Spanish	7	18	3	8	10	25	3	8	15	38	2	5	40	
Could not latch?	English	25	27	21	23	13	14	6	7	22	24	4	4	91	*Χ*^2^ (4, 131) = 9.141, *p* = .104
	Spanish	6	15	5	13	8	20	8	20	10	25	3	8	40	
Ineffective suck?	English	33	36	19	21	16	17	6	7	14	15	4	4	92	*Χ*^2^ (5, 132) = 1.113, *p* = .953
	Spanish	13	33	6	15	8	20	3	8	8	20	2	5	40	
Was there biting?	English	0	0	1	1	18	20	10	11	54	59	9	10	92	*Χ*^2^ (5, 132) = 3.885, *p* = .566
	Spanish	1	3	1	3	6	15	5	13	25	63	2	5	40	
Were they irritable in association with feeding?	English	24	26	17	18	19	21	6	7	24	26	2	2	92	*Χ*^2^ (5, 132) = 10.659, *p* = .059
	Spanish	4	10	4	10	10	25	2	5	20	50	0	0	40	
Was there vomiting?	English	20	19	27	26	15	14	19	18	23	22	1	1	105	*Χ*^2^ (5, 156) = 4.471, *p* = .484
	Spanish	7	14	11	22	11	22	6	12	16	31	0	0	51	
Was there arching?	English	14	13	20	19	29	27	10	9	26	25	7	7	106	*Χ*^2^ (5, 157) = 8.824, *p* = .116
	Spanish	4	8	7	14	9	18	3	6	24	47	4	8	51	
Did they show excessive movements?	English	16	15	15	14	27	25	9	8	32	30	7	7	106	*Χ*^2^ (5, 157) = 8.558, *p* = .128
	Spanish	3	6	6	12	9	18	3	6	27	53	3	6	51	
Was there difficulties maintaining or regulating proper body temperature?	English	4	4	7	7	12	11	7	7	58	55	18	17	106	*Χ*^2^ (5, 157) = 14.686, *p* = .012
	Spanish	0	0	2	4	9	18	2	4	38	75	0	0	51	
Was there difficulty sleeping?	English	43	44	13	13	18	18	8	8	16	16	0	0	98	*Χ*^2^ (5, 149) = 6.226, *p* = .183
	Spanish	14	27	6	12	9	18	7	14	15	29	0	0	51	
*Infancy Questions*															
Were they happy in the first 12 months of their life?	English	31	29	55	51	17	16	3	3	0	0	1	1	107	*Χ*^2^ (5, 162) = 3.271, *p* = .514
	Spanish	19	35	31	56	4	7	1	2	0	0	0	0	55	
Were they placid in the first 12 months of their life?	English	9	9	34	32	22	21	16	15	20	19	4	4	105	*Χ*^2^ (5, 160) = 23.817, *p* = .001
	Spanish	18	33	23	42	8	15	3	5	3	5	0	0	55	
Were they easy going in the first 12 months of their life?	English	22	21	51	48	16	15	14	13	4	4	0	0	107	*Χ*^2^ (5, 162) = 10.363, *p* = .035
	Spanish	16	29	19	35	9	16	3	5	8	15	0	0	55	
Were they affectionate in the first 12 months of their life?	English	22	21	48	45	20	19	6	6	9	8	1	1	106	*Χ*^2^ (5, 161) = 9.012, *p* = .109
	Spanish	22	40	16	29	9	16	5	9	3	5	0	0	55	
Were there any difficulties with suck/swallow?	English	26	24	21	20	29	27	8	7	21	20	2	2	107	*Χ*^2^ (5, 161) = 9.641, *p* = .086
	Spanish	9	17	10	19	9	17	4	7	22	41	0	0	54	
Were there any difficulties with failure to gain weight?	English	18	17	18	17	21	20	2	2	48	45	0	0	107	*Χ*^2^ (4, 161) = 7.446, *p* = .114
	Spanish	7	13	9	17	7	13	6	11	25	46	0	0	54	
Were there any reflux/gastro/oesophageal problems?	English	38	36	26	24	16	15	14	13	11	10	2	2	107	*Χ*^2^ (5, 161) = 21.802, *p* = .001
	Spanish	9	17	13	24	11	20	1	2	19	35	1	2	54	
Were there any difficulties with transitioning to solid food?	English	12	11	15	14	22	21	12	11	43	40	3	3	107	*Χ*^2^ (5, 161) = 4.723, *p* = .451
	Spanish	8	15	6	11	7	13	10	19	23	43	0	0	54	
Were there any difficulties with asthma/wheezing?	English	3	3	2	2	13	12	9	8	79	74	1	1	107	*Χ*^2^ (5, 161) = 3.908, *p* = .563
	Spanish	0	0	3	6	5	9	5	9	41	76	0	0	54	
Were there any difficulties with coughing?	English	1	1	5	5	17	16	17	16	65	61	1	1	106	*Χ*^2^ (5, 160) = 0.968, *p* = .965
	Spanish	1	2	2	4	8	15	8	15	35	65	0	0	54	
Were there any difficulties with pneumonia?	English	1	1	0	0	9	8	11	10	86	80	0	0	107	*Χ*^2^ (5, 161) = 4.268, *p* = .234
	Spanish	0	0	0	0	5	9	1	2	48	89	0	0	54	
Were there any difficulties with bronchitis?	English	1	1	1	1	12	11	9	8	84	79	0	0	107	*Χ*^2^ (5, 161) = 3.505, *p* = .477
	Spanish	1	2	0	0	7	13	9	17	37	69	0	0	54	
Was there difficulty sleeping?	English	38	37	19	19	29	28	8	8	8	8	0	0	102	*Χ*^2^ (4, 156) = 6.111, *p* = .191
	Spanish	14	26	8	15	15	28	7	13	10	19	0	0	54	

## Discussion

The GASR was translated into Spanish, Traditional Chinese, Italian and Hindi. After completion of the community and final proofreading steps, acceptable translations were obtained and made available to the Angelman community. During our experience of managing and governing the translation and proofreading project, we refined the process to reduce burden on the research team and our proofreaders for future translations utilising crowdsourcing [17]. These relate to the process of proofreading, and management of translation projects.

With respect to establishing translation project for future languages, our first step is to source machine translations to create the initial language translation on *Crowdin*. The second step is to break the registry content into individual tasks based on word count and create a document with (1) links to the *Crowdin* editor for each task, (2) task name and description, and (3) task word count. The third step is to administer training covering completing tasks in the editing tool, and access to the in-context tool for reviewing content within the website to proofreaders. The translation projects would continue to be managed by the author (MT). Further to this, greater efforts would be made to validate the translations generated. An initial validation of the Newborn and Infancy module of the registry was promising, with few differences between English and Spanish responses.

### Potential limitations to the crowdsourcing approach

There were two possible limitations identified in the current study. These limitations relate to the translations, but may also be relevant to validation testing.

#### Participants were time poor

In some cases, participants were unavailable to complete translation tasks due to competing priorities and responsibilities. Future strategies to assist families may include recruiting a larger number of proofreaders, facilitating support and connection between proofreaders, ensuring that larger proofreading tasks are broken down into smaller chunks, and providing incentives such as a donation to their local Angelman organization.

#### Community proofreaders were difficult to source for some languages

As participation in the registry was very low for some regions, such as Asia, Africa and the Middle East, it was difficult to source proofreaders for languages spoken in these regions such as Arabic or Hindi. As a result, the team opted for professional translation via vendors who can integrate with *Crowdin*, with Crowdsourcing reserved for registry revisions once families have become more engaged for the Hindi language.

## Conclusion

Crowdsourcing was an effective tool for upgrading translations, facilitating proofreading and integrating translated versions of the Global Angelman Syndrome Registry on an online platform. The availability of translations has led to greater participation and engagement of Angelman populations from regions where Spanish, Italian, Traditional Chinese and Hindi are spoken. The use of Crowdsourcing via online translation software such as *Crowdin* helps to manage ongoing translation and proofreading needs for research projects and maintain community participation and buy-in. However, further efforts are needed beyond translation to validate the translation of the registry for different communities.

## Data Availability

Data from the Global Angelman Syndrome Registry is available upon request, subject to ethical and legal safeguards. Interested parties can request access to the data by submitting a request form at: https://www.angelmanregistry.info/registry-data-request/. Alternately, a deidentified data set is currently being made available by the Critical Path Institute: https://c-path.org/programs/rdca-dap/overview/platform/. Please email curator@angelmanregistry.info or datarequest@angelmanregistry.info for more information.
